# Response to "Eleutheroembryo-based new approach methodologies for endocrine and metabolic endpoints: a comment on Christou et al. (2026)"

**DOI:** 10.1016/j.namjnl.2026.100131

**Published:** 2026-09-11

**Authors:** Maria Christou, Anastasia Georgantzopoulou, Maria Hultman, You Song, Aina Charlotte Wennberg, Sophie Mentzel, Michelle Embry, Amelie Ott, Takahiro Suzuki, Giacomo Grassi, Kyle Roush, Adam Lillicrap

**Affiliations:** a Norwegian Institute for Water Research (NIVA), Oslo, Norway; b Health and Environmental Sciences Institute, Washington DC, USA; c International Collaboration on Cosmetics Safety (ICCS), NY, USA; d Kao Corporation, R&D Safety Science Research, Tochigi 321-3497, Japan; e L’Oréal, Paris, France; f Procter & Gamble, Cincinnati, Ohio, USA

Dear Editor-in-Chief,

We thank Gregory F. Lemkine and colleagues for their thoughtful and constructive comments regarding our review, "The rise of new approach methodologies (NAMs) for environmental safety assessment of chemicals: a systematic review of the current status, challenges and strategies for broader adoption of NAMs." Their letter highlights important developments within the field of endocrine-related NAMs and provides additional context regarding eleutheroembryo-based assays that may be valuable for readers of the review.

We are pleased that the authors recognise both the objectives and overall conclusions of our review, and we welcome the opportunity to clarify several aspects according to their comments and our interpretations.

## Scope of the review

As noted in both our review and herein, endocrine disruption was not included within the primary scope of the systematic review, but we did not explicitly **exclude** endocrine endpoints as stated by Lemkine et al. On the contrary, we stated “alternative approaches for assessing chemicals causing endocrine disruption as an endpoint were not the focus of this study as these have previously been reviewed extensively by Mitchell et al. (2023) **but were nevertheless registered in the database of aquatic toxicity testing**.” This allowed us to focus on other areas of NAMs for environmental safety assessments of chemicals where no equivalent synthesis was currently available.

We agree that endocrine endpoints continue to appear throughout the review because endocrine activity intersects with several broader discussions, including endpoint coverage, embryo models, knowledge gaps, and future research priorities. Consequently, while endocrine disruption was not a focal topic, it could not be entirely separated from the broader environmental NAM landscape. Similarly, invertebrate NAMs were not the focus of the review, but were still referred to in our article, specifically in Table 3. Importantly, Table 3 was developed to illustrate the different types of NAM readiness levels – not to be an exhaustive list of all methods, OECD TGs, etc. The caption of Table 3 is “Examples of different methods at different levels of readiness from reviewed literature database (at the time of publishing this article).” So, including all methods at NAMRL-8 does not fit with the purpose of Table 3 or the article. Table 3 highlights that at the time of the review there were only a very small number of environmental NAMs that had reached a NAMs readiness level of 9 (i.e., method accepted for regulatory test requirements e.g. EU REACH).

## OECD-test guideline eleutheroembryo assays

We appreciate the authors' detailed overview of OECD Test Guidelines 248 (XETA), 250 (EASZY), 251 (RADAR), and 252 (REACTIV). These assays represent significant scientific and regulatory achievements and demonstrate how mechanistically informed organism-based methods can progress through validation and international standardisation processes.

We agree that, when considered individually within the readiness framework presented in our review, these methods would satisfy the criterion corresponding to methods formally adopted by an international standardisation body (i.e., NAMs readiness level 8). Their omission from Table 3 was not due to an assessment that they lacked sufficient maturity but rather reflects the broader scoping decision to exclude endocrine disruption NAMs from the principal analysis. The authors are therefore correct that readers focusing specifically on endocrine endpoints may not obtain a complete picture of currently standardised endocrine-related NAMs from our review alone. We welcome this clarification and consider their letter a useful complement to our original publication.

At the same time, we note that inclusion of these assays would not materially alter one of the central conclusions of our review: namely, that while regulatory acceptance of environmental NAMs is increasing, only a relatively limited number of methods have currently progressed through formal validation and standardisation pathways compared with the much larger number of methods still in development. Indeed, the examples highlighted by Lemkine et al. provide further evidence that advancement to high readiness levels is achievable and it is promising that regulatory uptake continues to expand.

## Emerging assays for thyroid and metabolic endpoints

We thank the authors for drawing attention to the TETHYS and EMERGE assays. These methods address areas that our review identified as comparatively underrepresented within the current environmental NAM landscape, particularly thyroid related and metabolism associated endpoints. However, neither of these methods were published at the time of our literature review (pre-November 2024). This highlights the most important comment to our review in that the field of environmental NAMs is at an extremely fast pace of development and reinforces our conclusion that targeted innovation is occurring in areas where coverage gaps have been recognised by both researchers and regulators. Furthermore, we acknowledge that the ATEST database, developed during the review, only offers a snapshot in time of environmental NAMs for environmental safety assessments of chemicals. Therefore, we welcome future opportunities to maintain and update the database to ensure it remains a useful repository for cataloguing emerging developments in the field of environmental NAMs.

## Search strategy and the human-keyword exclusion

The authors raise an important methodological question regarding the exclusion of records containing the term "human". The purpose of this filter was to reduce retrieval of studies focused exclusively on human biomedical, pharmaceutical, or clinical applications that were outside the environmental assessment context addressed by the review. It was not intended to exclude environmental studies employing aquatic model species or approaches that make reference to conserved human orthologues.

As with all systematic reviews, search strategies involve a balance between sensitivity and specificity. We acknowledge the possibility that a small number of environmentally relevant studies may have been excluded inadvertently during the screening process if terminology related to human orthologues featured prominently in titles, abstracts, or keywords. The point raised by the authors is therefore valuable and will be considered in future updates or expansions of the database.

Importantly, our broader conclusions regarding endpoint coverage, readiness levels, and barriers to adoption were derived from several thousand screened studies and multiple lines of evidence. Consequently, we do not believe that inclusion of the specific examples cited would substantially change the overall findings of the review but would have made the screening exponentially longer.

## Concluding remarks

We thank Gregory F. Lemkine and co-authors for their constructive engagement with our work. Their letter highlights notable progress in endocrine-related eleutheroembryo assays, including several OECD-adopted methods and promising emerging approaches that warrant continued attention from the environmental toxicology community.

We view these observations as complementary to, rather than contradictory to, the conclusions of our review. Together, the review and the accompanying correspondence emphasize that environmental NAMs are advancing rapidly, that regulatory standardisation is increasingly achievable and that continued innovation in areas such as endocrine and metabolic disruption will further strengthen next-generation chemical safety assessment.

## Ethics declaration

The author(s) declare(s) that the study does not involve humans nor animal subjects.

## CRediT authorship contribution statement

**Maria Christou:** Formal analysis, Investigation, Methodology, Writing – review & editing, Writing – original draft. **Anastasia Georgantzopoulou:** Data curation, Formal analysis, Methodology, Investigation, Writing – review & editing. **Maria Hultman:** Investigation, Methodology, Formal analysis, Data curation, Writing – review & editing. **You Song:** Data curation, Formal analysis, Methodology, Investigation, Writing – review & editing. **Aina Charlotte Wennberg:** Data curation, Formal analysis, Visualization, Writing – review & editing. **Sophie Mentzel:** Visualization, Writing – review & editing. **Michelle Embry:** Conceptualization, Data curation, Formal analysis, Methodology, Investigation, Writing – review & editing. **Amelie Ott:** Conceptualization, Funding acquisition, Writing – review & editing. **Takahiro Suzuki:** Writing – review & editing. **Giacomo Grassi:** Writing – review & editing. **Kyle Roush:** Writing – review & editing. **Adam Lillicrap:** Conceptualization, Funding acquisition, Investigation, Methodology, Project administration, Resources, Supervision, Writing – original draft, Writing – review & editing.

## Declaration of competing interest

The authors declare that they have no known competing financial interests or personal relationships that could have appeared to influence the work reported in this paper.

## Data Availability

No data was used for the research described in the article.

